# KRAS Mutations as Predictive Biomarkers for First-Line Immune Checkpoint Inhibitor Monotherapy in Advanced NSCLC: A Systematic Review and Meta-Analysis

**DOI:** 10.3390/curroncol32060365

**Published:** 2025-06-19

**Authors:** Filip Marković, Jelena Milin-Lazović, Nikola Nikolić, Aleksa Golubović, Mihailo Stjepanović, Milica Kontić

**Affiliations:** 1 Clinic for Pulmonology, University Clinical Centre of Serbia, 11000 Belgrade, Serbia; filip.markovic@kcs.ac.rs (F.M.); aleksa.golubovic@kcs.ac.rs (A.G.); mihailo.stjepanovic@kcs.ac.rs (M.S.); milica.kontic@med.bg.ac.rs (M.K.); 2 Department for Medical Statistics and Informatics, School of Medicine, University of Belgrade, 11000 Belgrade, Serbia; jelena.milin@med.bg.ac.rs; 3 School of Medicine, University of Belgrade, 11000 Belgrade, Serbia

**Keywords:** KRAS, NSCLC, predictive biomarker, immune checkpoint inhibitors, meta-analysis

## Abstract

Recent research suggests a link between KRAS mutations and the effectiveness of ICIs, as KRAS-driven tumors may possess unique immunogenic features that influence the tumor microenvironment. These mutations can increase tumor mutation burden (TMB) and neoantigen load, potentially leading to improved responses to ICIs. This meta-analysis aims to consolidate existing evidence on the impact of KRAS mutations as a predictive factor for survival and treatment outcomes in patients with advanced NSCLC treated with ICIs. A comprehensive search strategy was designed by a biostatistician and pulmonologist, targeting PubMed, Web of Science, and Scopus databases up to May 2022. The outcomes assessed included overall survival (OS) and progression-free survival (PFS), reported as log hazard ratios (HRs) with corresponding standard errors (SEs). A pooled estimate of the HR effect size was calculated using Review Manager (RevMan, Cochrane Collaboration, London, UK). Heterogeneity among studies was evaluated using the Cochran Q test and the I^2^ statistic. Ultimately, 10 articles were deemed suitable for inclusion in the systematic review from a total of 8722 screened titles and abstracts. The presence of KRAS+ mutations had a significant prognostic factor for better OS in NSCLC patients treated with checkpoint inhibitors (HR = 0.89, 95% CI: 0.79–0.99) and for better PFS in NSCLC patients treated with checkpoint inhibitors (HR = 0.72, 95% CI: 0.59–0.87). In conclusion, our study indicates that KRAS mutations may serve as a potential positive predictive biomarker in patients with advanced non-small-cell lung cancer treated with immune checkpoint inhibitor monotherapy.

## Simple Summary

This meta-analysis investigates whether KRAS mutations can predict better outcomes in patients with advanced non-small-cell lung cancer (NSCLC) treated with immune checkpoint inhibitors (ICIs). By reviewing 10 studies selected from a total of 8722 screened, the results show that KRAS mutations are associated with improved overall survival (OS) and progression-free survival (PFS) in these patients. These findings suggest that KRAS mutations may serve as a useful biomarker to identify NSCLC patients who are more likely to benefit from ICI monotherapy.

## 1. Introduction

The treatment landscape for advanced non-small-cell lung cancer (NSCLC) has been profoundly transformed by the advent of immune checkpoint inhibitors (ICIs). These therapies, which aim to reactivate the host immune response against tumor cells, have shown significant survival benefits across multiple cancer types, including NSCLC [1].

However, despite these advances, not all patients benefit equally. A substantial proportion of NSCLC patients demonstrate primary resistance to ICIs, and many develop acquired resistance over time. This variability underscores the critical need for robust predictive biomarkers that can help identify which patients are most likely to respond [1,2].

KRAS is among the most commonly mutated genes in NSCLC, especially in adenocarcinomas, and contributes to tumor progression and metabolism [3]. Emerging evidence links KRAS mutations to ICI efficacy, potentially due to their impact on tumor immunogenicity and the microenvironment [4]. KRAS-driven tumors can promote immune evasion by activating downstream pathways and releasing immunosuppressive cytokines, which attract regulatory cells and exclude effector immune cells [5]. These tumors often display high PD-1 expression and increased CD8+ T-cell infiltration, features of an inflamed microenvironment. As KRAS mutations are more frequent in smokers—who typically have higher tumor mutational burden and neoantigen load—this may further support ICI responsiveness [4,6]. Still, the mechanisms by which KRAS shapes the immune landscape remain unclear and warrant further investigation.

Despite these insights, the role of KRAS mutations as a predictive biomarker for ICI therapy remains controversial and underexplored. Some studies report that NSCLC patients harboring KRAS mutations may experience improved outcomes with ICI therapy compared to chemotherapy [7,8].

KRAS G12C is the most common KRAS mutation found in NSCLC, representing approximately 40% of all KRAS-mutant cases. This specific alteration has garnered significant attention in recent years with the development of targeted therapies, such as sotorasib and adagrasib, which selectively inhibit the KRAS G12C mutant protein. However, despite their clinical promise, these targeted agents are currently approved only for use in the second-line or later settings following disease progression on standard therapies [9,10].

As a result, the initial, or first-line, treatment approach for patients with advanced NSCLC harboring KRAS mutations—including KRAS G12C—remains aligned with that for patients lacking actionable oncogenic drivers. These patients are typically managed according to PD-L1 expression status; those with high PD-L1 expression (≥50%) may receive immune checkpoint inhibitor (ICI) monotherapy, while others are generally treated with a combination of ICIs and platinum-based chemotherapy [10]. Despite the availability of KRAS-targeted therapies in later lines, the decision-making for first-line treatment in KRAS-mutant NSCLC is still primarily guided by PD-L1 expression and clinical factors, rather than KRAS mutation status itself.

KRAS mutations have been associated with adenocarcinoma histology, smoking history, higher tumor mutation burden (TMB) and programmed cell death ligand 1 (PD-L1) expression [11]. Given the biological rationale and evolving clinical evidence, it is hypothesized that KRAS mutations could serve as a predictive marker for selecting patients who may benefit most from first-line ICI monotherapy. Several studies have examined this question, but results are inconsistent, and no consensus has been established [11]. This lack of consensus highlights the need for a systematic and quantitative synthesis of the available data. A clearer understanding of the predictive value of KRAS mutations could have important clinical implications, helping to refine treatment strategies and guide biomarker-driven decision-making in the first-line setting.

This meta-analysis aims to synthesize available evidence and evaluate the predictive value of KRAS mutations for the efficacy of first-line ICI monotherapy in patients with advanced NSCLC. By thoroughly examining existing studies, we aim to provide a more definitive understanding of KRAS mutations as biomarkers. This insight could lead to better, more personalized treatment approaches, improving how clinicians manage advanced NSCLC [12].

## 2. Method

A systematic review was conducted in accordance with the Preferred Reporting Items for Systematic Reviews and Meta-Analyses (PRISMA) guidelines [13] and the Meta-analysis of Observational Studies in Epidemiology (MOOSE) guidelines [14]. A standardized protocol was developed specifically for this review and implemented by independent reviewers. This protocol has not been registered. We included studies conducted in humans that reported survival outcomes—specifically overall survival (OS) and/or progression-free survival (PFS)—related to the use of immune checkpoint inhibitors in non-small-cell lung cancer (NSCLC). Eligible studies comprised phase II and phase III clinical trials, which provide the most robust evidence on treatment efficacy and survival. Phase I trials, primarily focused on safety and dose escalation, were excluded.

In addition to clinical trials, we also considered real-world data (RWD) studies that met our predefined inclusion criteria. In cases where the same study was reported in multiple publications (e.g., interim vs. final results), we included the most recent publication with the longest available follow-up to ensure data completeness and avoid duplication.

The screening process for systematic review inclusion transpired in two distinct phases, with any disparities resolved through discussion, the intervention of a third reviewer, or by consensus. Inclusion criteria for the first phase for screening titles and abstracts were as follows: Study type: original research (cross sectional, observational studies, case control, RTC); Population: Non-small-cell lung carcinoma. NSCLC, Squamous cell carcinoma, Adenocarcinoma, Large-cell carcinoma; Intervention: check point inhibitors (alone or in comparison with chemotherapy or targeted therapy or other types of check point inhibitors); Outcome: overall survival, progression-free survival, disease-free survival. Exclusion criteria for the first phase for screening titles and abstracts were as follows: 1. Studies published in a foreign language; 2. Publications that are not original research articles, such as reviews, meta-analyses, systematic reviews, commentaries, editorials, conference abstracts, case reports, or registration studies; 3. Studies involving the wrong population, including animal or cell line research, other tumor types, or unrelated diseases. 4. Studies in which patients were not treated with checkpoint inhibitors. In next phase of full text screening, studies were included based on the following criteria [1]: studies involving patients with non-small-cell lung cancer [2], studies in which patients were treated (in the first line) with checkpoint inhibitors, and [3] studies in which the influence of KRAS +/− status was examined on OS or PFS. Exclusion criteria comprised studies involving (1) other cancers, (2) studies without survival outcomes (either overall or progression-free), or (3) studies lacking key data for extraction.

### 2.1. Search Strategy

A sensitive search strategy was developed by a biostatistician (J.M.L) and a pulmonologist (M.K.), targeting PubMed, Web of Science, and Scopus databases until May 2022. The following keywords were used to search the databases: (“Immune-checkpoint inhibitor” OR “PD-1” OR “PD-L1” OR “Pembrolizumab” OR “Nivolumab” OR “Atezolizumab” OR “Avelumab” OR “Durvalumab” OR “CTLA-4” OR “Ipilimumab” OR “Tremelimumab”) AND (“Non-small-cell lung carcinoma” OR “NSCLC” OR “Squamous cell carcinoma” OR “Adenocarcinoma” OR “Large cell carcinoma”) AND (“survival” OR “overall survival” OR “survival analysis” OR “progression-free survival”).

Manual searches of reference lists, relevant reviews, editorials, and consultations with field experts were also conducted.

### 2.2. Article Screening and Selection

Three independent reviewers (F.M, N.N, and A.G) assessed the eligibility of titles and abstracts, proceeding to full-text screening based on predefined criteria. Disagreements were resolved through consensus (F.M, N.N, and A.G) or arbitration (J.M.L, M.K.).

### 2.3. Data Abstraction and Quality Assessment

Three reviewers (F.M, N.N, and A.G) independently extracted data including author name, publication year, country, patient numbers, study design, inclusion/exclusion criteria, TNM stage, and relevant markers. Survival outcome data, encompassing OS, PFS, Hazard Ratios (HR), and 95% confidence intervals (95% CI), were also extracted.

### 2.4. Risk of Bias

The risk of bias in individual studies was assessed using criteria proposed by the GRADE Working Group, focusing on eligibility criteria, measurement quality, confounding control, and follow-up completeness [15]. Three reviewers (F.M, N.N, and A.G) independently evaluated bias within and across studies using an adapted version of the Newcastle–Ottawa tool for observational studies [16].

### 2.5. Statistical Analysis

Outcomes were evaluated based on overall survival (OS) and progression-free survival (PFS), expressed as the logarithm of the hazard ratio (log HR) and corresponding standard error (SE). For studies that did not report HRs and 95% confidence intervals (CIs) directly [17,18], log HR and SE were derived by extracting survival data from Kaplan–Meier curves using Web Plot Digitizer v4.4 [19]. Hazard ratios were subsequently estimated using the method described by Tierney et al. [20]. When the number of patients at risk was not explicitly provided, we extracted this information when possible or estimated it based on the total number of patients included in the survival analysis and selected time points, with adjustments made to account for censored data. To derive the summary HR effect size, we pooled individual trial results using Review Manager, version 5.3, from the Cochrane Collaboration. Heterogeneity was assessed through the Cochran Q test and I^2^ statistic, with heterogeneity defined as I^2^ > 50% or a *p*-value < 0.10, following Higgins and Thompson [21]. A fixed-effect model was used for all analyses, given the consistently low heterogeneity across studies [21]. Study weights were calculated using the inverse variance method, adjusted according to the applied effect model, to determine each study’s contribution to the pooled hazard ratio (HR). Sensitivity analyses were conducted to assess the potential impact of extracting survival data from Kaplan–Meier curves. Forest plots were generated for each analysis, displaying individual HRs (boxes), 95% confidence intervals (horizontal lines), and study weights (reflected by box size); the pooled effect estimate was represented by a diamond. Publication bias was evaluated using a linear regression test for funnel plot asymmetry, with a *p*-value < 0.05 considered statistically significant. We used EZR software (https://www.jichi.ac.jp/usr/hema/EZR/statmedEN.html accessed on 14 June 2025) for meta-analysis.

## 3. Results

**Figure 1:**
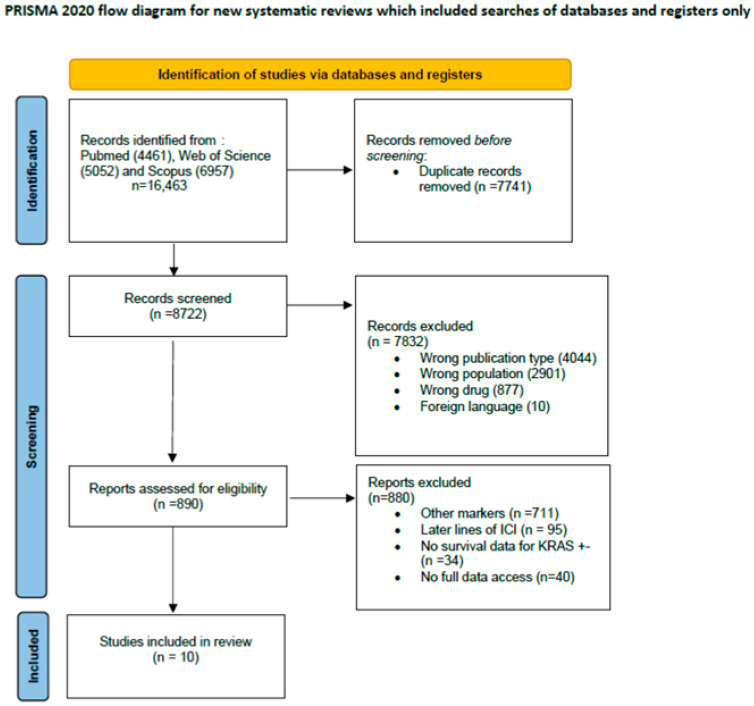
Study selection process.

A comprehensive search yielded a total of 16,463 potentially eligible articles. Following the elimination of duplicate entries, 8722 titles and abstracts were screened. Within the phase of reading titles and abstracts, 7822 articles were excluded after careful examination due to incorrect publication type (*n* = 4044; systematic review, review, case reports, conference abstrackrasts), irrelevant population (*n* = 2901; animal, cells, other tumors), or wrong drugs (*n* = 877; not check point inhibitors). Among the 890 articles subjected to full-text review, 880 were further excluded due to other markers (*n* = 711), later lines of ICI (*n* = 95), absence of survival data KRAS +/− (*n* = 34), or unavailability of the full-text version (*n* = 40). Ultimately, 10 articles were deemed suitable for inclusion in the systematic review, 8 with OS data, 2 with PFS data, and 3 with both OS and PFS data. A detailed description of the study selection process is visually presented in Figure 1.

The characteristics of all 10 publications [17,18,22,23,24,25,26,27,28,29] included in the systematic review are presented in detail in Table 1. All studies were retrospective, and most of the studies were conducted in Europe [5,23,24,25,27,28], four in the USA [17,18,22,29], and one in China [25]. The studies were published between 2019 and 2022, with sample sizes ranging from 37 [28] to 705 patients [29]. Six studies included only patients with metastatic disease (stage IV), while four studies included patients with both advanced and metastatic diseases (stages III and IV). The longest follow up was 34 months [18]. Eight studies analyzed KRAS mutations, and two studies specifically assessed KRAS G12C mutation in NSCLC patients. Most studies did not report the method for KRAS mutation assessment.

### 3.1. KRAS +/− Status and Overall Survival

**Figure 2:**
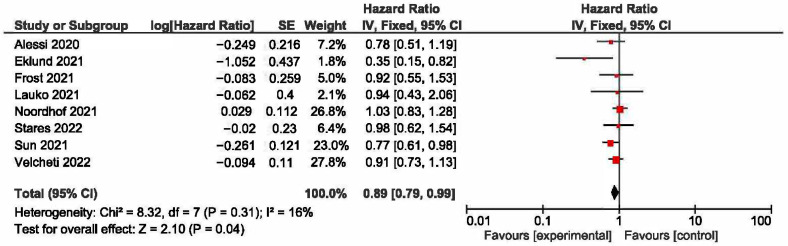
KRAS +/− status and overall survival [17,18,22,24,26,27,28,29].

**Figure 3:**
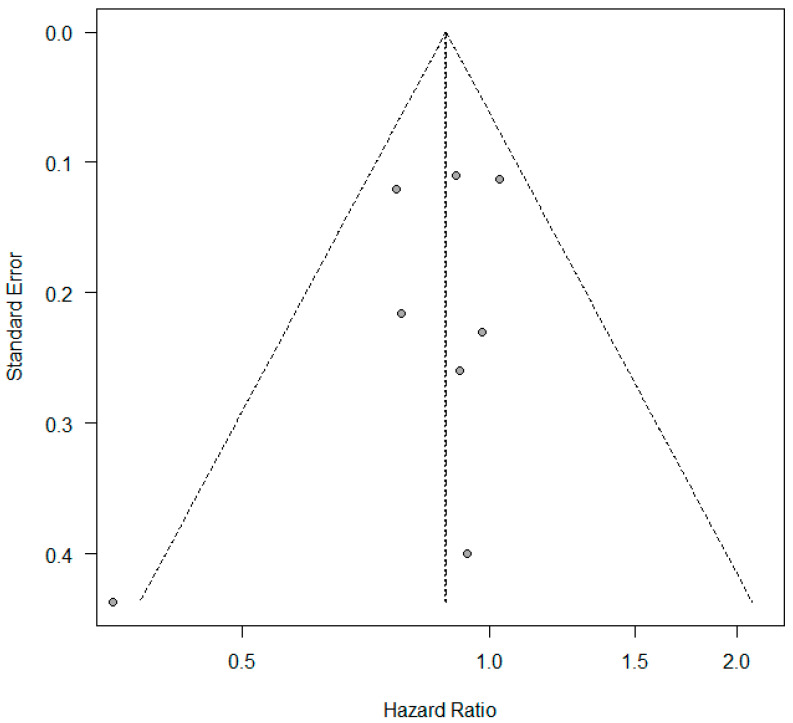
Funnel plot—studies with overall survival.

A meta-analysis was conducted to evaluate the association between KRAS mutations and overall survival (OS) in NSCLC patients receiving first-line immune checkpoint inhibitor (ICI) monotherapy. A total of eight studies had OS as an outcome. The presence of KRAS mutations was a significant prognostic factor for better OS in NSCLC patients treated with checkpoint inhibitors (HR = 0.89, 95% CI: 0.79–0.99) (Figure 2). There was a low degree of heterogeneity in the OS analysis (I^2^ = 16%) and no publication bias (*p* = 0.286). The sensitivity analysis, excluding two studies [17,18] that extracted survival rates from Kaplan–Meier curves, showed a non-significant HR (HR = 0.87, 95% CI: 0.76–1.0). The funnel plot for studies with overall survival is presented in Figure 3.

### 3.2. KRAS +/− Status and Progression-Free Survival

**Figure 4:**
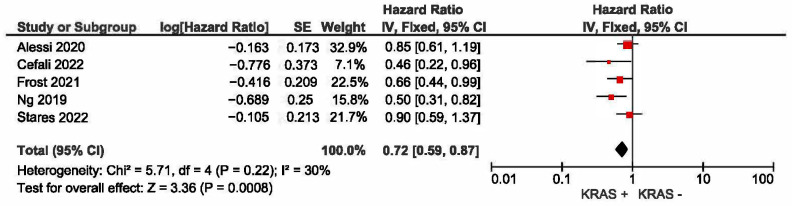
KRAS +/− status and progression-free survival [22,23,24,25,27].

**Figure 5:**
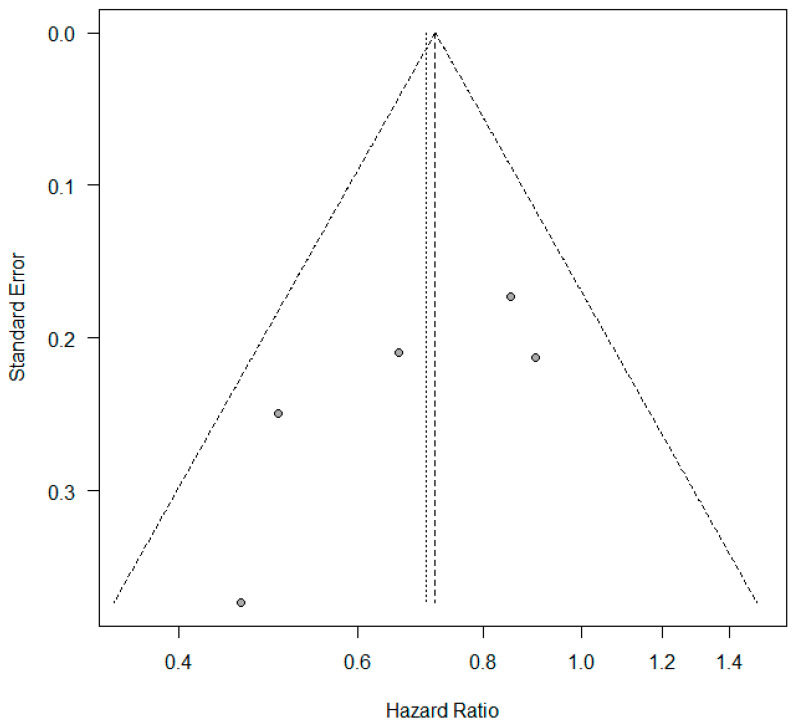
Funnel plot—studies with progression-free survival.

A meta-analysis was conducted to evaluate the association between KRAS mutation status and progression-free survival (PFS) in patients with non-small-cell lung cancer (NSCLC) receiving first-line immune checkpoint inhibitor monotherapy. A total of five studies had PFS as an outcome. The presence of KRAS mutations was a significant prognostic factor for better PFS in NSCLC patients treated with checkpoint inhibitors (HR = 0.72, 95% CI: 0.59–0.87) (Figure 4). There was a low degree of heterogeneity in the PFS analysis (I^2^ = 30%) and no publication bias (*p* = 0.124). The funnel plot for studies with progression-free survival is presented in Figure 5.

## 4. Discussion

The findings from this comprehensive meta-analysis offer pivotal insights into the predictive value of KRAS mutations in determining the efficacy of first-line ICIs in patients with NSCLC.

While previous studies have presented mixed results, our analysis delineates clearer patterns of response based on KRAS mutational status. Consistent with some prior research, our findings suggest that patients with KRAS mutations exhibit better outcomes in terms of overall and progression-free survival with ICIs compared to their KRAS wild-type counterparts.

KRAS is the most frequently mutated oncogene in NSCLC, with mutations present in 30% of lung adenocarcinomas and 5% of squamous cell carcinomas [30]. KRAS mutations (KRASm) have historically been difficult to target, and while a second-line targeted therapy for KRAS G12C NSCLC inhibitors like sotorasib and adagrasib are now approved, standard frontline treatment of KRASm metastatic NSCLC still consists of immunotherapy alone or in combination with chemotherapy [31]. While sotorasib and adagrasib offer modest survival benefits in the second line of therapy, both are being studied in combination with other therapies to enhance efficacy and overcome resistance. Additionally, next-generation KRAS inhibitors, including D-1553, IBI351, and JDQ443, have shown promising results in early-phase I/II trials. Ongoing studies exploring combinations with SHP2, FAK, EGFR inhibitors, and immunotherapy—especially in the first-line setting—aim to further improve patient outcomes and combat resistance [32].

The extent to which KRAS mutational status informs currently available frontline therapeutic strategies for metastatic NSCLC remains an unresolved and critical question.

KRAS mutations, unlike EGFR and ALK, are associated with a history of smoking, high PD-L1 expression, and a high tumor mutational burden (TMB). As a result, this has led to the idea that patients with advanced NSCLC who carried KRASm could respond to ICIs more favorably than those with KRAS wild-type [30,33].

Some studies in the second line and beyond setting have suggested a better overall and progression-free survival for KRAS mutated patients treated with immunotherapy compared to KRAS wild-type patients [34,35]. They implied that patients with KRASm might have better response to ICI due to a larger proportion of smokers and a tumor environment with a predominance of immunological cells because of activation of KRAS signaling pathways as well as higher TMB [28].

However, whether patients with NSCLC and positive KRASm have a better OS compared to KRAS wild-type patients when treated with first-line ICI remains unclear, as previous studies gave inconclusive results [36].

A meta-analysis of randomized trials (Keynote, Checkmate, OAK, Poplar) performed by Landre T. et al. showed that KRAS is a good predictive biomarker for survival, both OS and PFS [37]. One of the possibilities for that result is that the patients with KRAS mutation have higher TMB and higher PD-L1 expression. Similar results were observed in retrospective studies done by Ng TL et al. [25], where researchers examined the impact of oncogene-driver subtype, PD-L1 status, and smoking status. They showed that PD-L1 was higher for patients with KRASm and positive smoking status. This is consistent with findings that smokers have higher PD-L1 and TMB and more somatic mutations, therefore, a better response to immune checkpoint inhibitors [25]. In contrast, a retrospective study done by Jeanson A et al. assessing the effectiveness of ICI in advanced NSCLC with 282 patients, of which 162 were harboring KRAS mutation, showed no significant benefit of ICI, nor PFS or OS [38].

There are not many comparable studies referring to real-world data on this matter, especially the first-line monotherapy with ICI. Our goal was to collect real-world studies that can give us results about the predictive significance of KRAS mutation in patients with advanced and metastatic NSCLC treated with first-line ICI monotherapy.

Our analysis did not account for the specific KRAS mutation subtypes or co-mutation status in patients with KRAS-mutant NSCLC. However, several studies have explored the impact of these molecular differences. Zhao et al. reported that patients with the KRAS G12D mutation had significantly longer overall survival compared to those with other KRAS variants (HR 0.09, 95% CI 0.01–0.68, *p* = 0.02). Conversely, KRAS G12V mutations were associated with a trend toward worse overall survival, although this did not reach statistical significance (HR 1.94, 95% CI 0.95–3.96, *p* = 0.068) [39]. Similarly, Sun et al. found that KRAS G12V was associated with resistance to immune checkpoint inhibitor (ICI) monotherapy, even in patients with PD-L1 expression ≥50%. In their cohort, patients harboring KRAS G12V had the shortest median overall survival (5.4 months) with ICI monotherapy, compared to 19.7 months for G12C and 18.3 months for G12D. Notably, among patients with high PD-L1 expression, KRAS G12V was the only subtype in which ICI monotherapy was less effective than platinum-based chemotherapy. These findings suggest that KRAS G12V may represent a negative predictive biomarker for ICI monotherapy response, potentially due to unique tumor microenvironmental or oncogenic signaling features [40].

A study performed by Skoulidis F. et al. [41] showed that STK11/LKB1 co-mutations are associated with inferior objective response rate with PD-1 blockade in KRAS-mutant non-squamous NSCLC. STK11/LKB1 genomic alterations are associated with primary resistance to PD-1 axis inhibitors in PD-L1 positive NSCLC with significantly shorter PFS and OS in such patients regardless of PD-L1 and KRAS mutation status. The same study showed that patients with TP53 co-mutations had similar response rate [41]. Other studies confirmed beforementioned results regarding STK11/LKB1 co-mutations [42,43,44], suggesting that LKB1-deficient tumor cells stimulate neutrophil recruitment through the production of cytokines and chemokines and changes in metabolic reprogramming, which lead to a worse prognosis in such patients [42,43].

Eklund E.A. et al. [28], in their study with 580 participating patients with NSCLC, showed that KRAS mutation is a negative prognostic factor for OS irrelevant of treatment options, with a significant negative factor when treated with platinum doublet ((*p* = 0.001) with median OS 9 months vs. KRASWT 14 months). In that study, there was a larger proportion of patients with high PD-L1 (over 50%) in the mutated KRAS population compared to KRAS wild-type (43.0% vs. 32.7%). When it comes to patients treated with ICI, there was significantly better response to ICI treatment for patients with mutated KRAS, with a median OS of 23 months vs. 9 months for patients with wild-type KRAS (*p* = 0.028). Surprisingly, patients with KRAS wild-type showed a worse response to pembrolizumab monotherapy than to platinum doublet chemotherapy [28].

There are some previous retrospective studies with different results when comparing KRAS mutated and KRAS wild-type patients treated with ICI [17,18,22,23,24,25,26,27,28,29]. We aimed to homogenize the obtained results and try to give a conclusive statement. There remains a need for randomized clinical trials to address this topic.

## 5. Conclusions

In our study, patients with KRAS mutations demonstrated better OS as well as PFS compared to patients without KRAS mutations.

Our analysis underscores the value of incorporating comprehensive genomic profiling into routine clinical practice. This approach enables more precise identification of patients who are likely to benefit from immune checkpoint inhibitors, ultimately improving treatment outcomes and survival. Integrating KRAS mutation status into clinical decision-making may serve as a paradigm for the use of other genomic biomarkers to guide immunotherapy strategies.

## Figures and Tables

**Table 1:** Overview of the literature on KRAS status and OS and PFS in patients on first-line treatment with checkpoint inhibitors.

Reference	Year	Checkpoint Inhibitor	Stage	Median Follow-Up	Marker	Method	Endpoints	KRAS+ (n)	KRAS− (n)	Total (n)
**Alessi JV** [22]	2020	Pembrolizumab	IV	14.8	KRAS	NR	OS, PFS	81	153	234
**Cefalì M** [23]	2022	Pembrolizumab	IIIB/C, IV	NR	KRAS G12C	NGS	PFS	11	33	44
**Eklund E.A.** [28]	2021	Pembrolizumab	IV	7 months	KRAS	FISH	OS	20	17	37
**Frost N** [24]	2021	Pembrolizumab	III, IV	26.4	KRAS G12C	NGS	OS, PFS	62	57	119
**Lauko A.** [17]	2021	Nivolumab, Pembrolizumab	IV	NR	KRAS	NR	OS	23	16	39
**Ng T. L.** [25]	2019	Nivolumab, Pembrolizumab, Atezolizumab	III, IV	7.1 months	KRAS	NGS, FISH	PFS	77	112	189
**Noordhof A. L** [26]	2021	Pembrolizumab	IV	19.1 months	KRAS	NR	OS	338	257	595
**Stares M** [27]	2022	Pembrolizumab	IV	20 months	KRAS	NR	OS, PFS	NR	NR	130
**Sun L.** [29]	2021	Not reported	IV	NR	KRAS	NR	OS	363	342	705
**Velcheti V** [18]	2022	Pembrolizumab	IIIB/C, IV	34 months	KRAS	NR	rwToT	164	166	330

NR—Not reported; KRAS—Kirsten rat sarcoma; NGS—Next generation sequencing; OS—Overall survival; PFS—Progression-free survival; rwToT—real-world time on treatment.

## Data Availability

The original contributions presented in this study are included in the article. Further inquiries can be directed to the corresponding author(s).
